# Promoting Personalized Reminiscence Among Cognitively Intact Older Adults Through an AI-Driven Interactive Multimodal Photo Album: Development and Usability Study

**DOI:** 10.2196/49415

**Published:** 2024-01-23

**Authors:** Xin Wang, Juan Li, Tianyi Liang, Wordh Ul Hasan, Kimia Tuz Zaman, Yang Du, Bo Xie, Cui Tao

**Affiliations:** 1 Department of Computer Science North Dakota State University Fargo, ND United States; 2 Department of Computer Systems and Software Engineering Valley City State University Valley City, ND United States; 3 School of Nursing The University of Texas at Austin Austin, TX United States; 4 School of Information The University of Texas at Austin Austin, TX United States; 5 D Bradley McWilliams School of Biomedical Informatics UTHealth Houston Houston, TX United States

**Keywords:** aging, knowledge graph, machine learning, reminiscence, voice assistant

## Abstract

**Background:**

Reminiscence, a therapy that uses stimulating materials such as old photos and videos to stimulate long-term memory, can improve the emotional well-being and life satisfaction of older adults, including those who are cognitively intact. However, providing personalized reminiscence therapy can be challenging for caregivers and family members.

**Objective:**

This study aimed to achieve three objectives: (1) design and develop the GoodTimes app, an interactive multimodal photo album that uses artificial intelligence (AI) to engage users in personalized conversations and storytelling about their pictures, encompassing family, friends, and special moments; (2) examine the app’s functionalities in various scenarios using use-case studies and assess the app’s usability and user experience through the user study; and (3) investigate the app’s potential as a supplementary tool for reminiscence therapy among cognitively intact older adults, aiming to enhance their psychological well-being by facilitating the recollection of past experiences.

**Methods:**

We used state-of-the-art AI technologies, including image recognition, natural language processing, knowledge graph, logic, and machine learning, to develop GoodTimes. First, we constructed a comprehensive knowledge graph that models the information required for effective communication, including photos, people, locations, time, and stories related to the photos. Next, we developed a voice assistant that interacts with users by leveraging the knowledge graph and machine learning techniques. Then, we created various use cases to examine the functions of the system in different scenarios. Finally, to evaluate GoodTimes’ usability, we conducted a study with older adults (N=13; age range 58-84, mean 65.8 years). The study period started from January to March 2023.

**Results:**

The use-case tests demonstrated the performance of GoodTimes in handling a variety of scenarios, highlighting its versatility and adaptability. For the user study, the feedback from our participants was highly positive, with 92% (12/13) reporting a positive experience conversing with GoodTimes. All participants mentioned that the app invoked pleasant memories and aided in recollecting loved ones, resulting in a sense of happiness for the majority (11/13, 85%). Additionally, a significant majority found GoodTimes to be helpful (11/13, 85%) and user-friendly (12/13, 92%). Most participants (9/13, 69%) expressed a desire to use the app frequently, although some (4/13, 31%) indicated a need for technical support to navigate the system effectively.

**Conclusions:**

Our AI-based interactive photo album, GoodTimes, was able to engage users in browsing their photos and conversing about them. Preliminary evidence supports GoodTimes’ usability and benefits cognitively intact older adults. Future work is needed to explore its potential positive effects among older adults with cognitive impairment.

## Introduction

As the proportion of older individuals rapidly grows, an increasing number of older individuals are becoming concerned about their physical and mental well-being [1]. Steptoe et al [2] found that a decline in health is associated with a negative psychological state. In addition, aging is closely associated with various psychosocial stress factors, such as loneliness, personal losses, and lower socioeconomic status [3]. These factors may increase the risk of developing mental health disorders. In recent years, increasing evidence suggests that psychological well-being could be a potential asset for healthy aging [4].

Reminiscence therapy stands as a profound approach, rooted in a deep understanding of cognitive and emotional processes, designed to elevate the psychological well-being of older adults. This therapy transcends the mere recall of life histories, encompassing both oral and written narratives, in a multisensory journey that engages sight, sound, taste, touch, and smell [5-7]. It extends beyond a mere collection of activities, encompassing the contemplation of photographs, immersion in music, and the sharing of narratives about pivotal life events [5-8]. The underlying success of reminiscence therapy lies in its capacity to stimulate long-term memory, playing a pivotal role in fostering overall well-being, an attribute particularly invaluable for those grappling with short-term memory challenges [9]. As individuals review and discuss evocative materials, including vintage videos, cherished photographs, or sentimental household artifacts, they embark on a path to not only retrieve memories but also enhance their self-esteem, nurture interpersonal skills, and enrich their psychosocial well-being [4,10]. This is rooted in the therapy’s ability to harness the profound impact of these sensory stimuli. Significantly, the American Psychological Association recognizes the therapeutic potential of reminiscence therapy, attesting to its ability to ameliorate mental health conditions, elevate mood, and mitigate agitation, especially among individuals coping with Alzheimer disease or dementia [5].

Research by Tam et al [4] indicates that reminiscence intervention not only produces positive effects among older adults with dementia but also benefits cognitively intact older adults. For instance, it reduces the depressive symptoms of cognitively intact older adults, significantly improves their life satisfaction, and promotes their self-esteem, psychological well-being, and happiness.

Reminiscence interventions, whether administered within health care facilities such as hospitals, assisted living communities, or nursing homes, or within the familial cocoon of private homes, are bolstered by the skillful orchestration of trained professionals and caregivers [5,7,11]. In health care settings, psychologists, social workers, and specialists in geriatric care often take the helm in conducting these interventions, drawing upon a wealth of materials that hold personal significance for older adults. The tailored application of these materials, including photos, videos, and cherished objects, becomes a key facet in rekindling memory and reminiscence. These interventions can be adjusted to individual or group settings, finely attuned to the unique needs and preferences of older adults [5,11,12]. In private residences, reminiscence interventions are executed with dedication by family members, caregivers, or volunteers [5]. They use similar materials as those found within health care facilities, including familial photographs and cherished keepsakes, as a conduit to memory stimulation and the initiation of reminiscing. Nevertheless, conducting these interventions at home can be challenging due to a shortage of human resources [11]. Caregivers and family members, juggling numerous responsibilities, may find their time and resources stretched thin, underscoring the need for innovative approaches, such as the one proposed in this study, to bridge this gap.

To overcome the challenges of limited resources and specialized training in performing reminiscence interventions, flexible and effective computer-based interventions are highly beneficial [13-15]. We designed, developed, and tested GoodTimes, a personalized interactive multimodal photo album mobile app for cognitively intact older adults. It can be used on smartphones and tablets, providing older adults with on-the-go access to their photos as well as those provided by family members. This intelligent digital photo album allows users to browse and query photos in various orders, including chronological order, by location, by event, or by specific family members. To enhance the reminiscing experience, a voice assistant (VA) interacts with the user, providing information about the picture, such as the family members in the picture, the special moment, and the location. The VA also asks questions, answers user questions, and responds to user comments, creating a setting where special memories can be remembered and enjoyed. To ensure the user’s comfort and avoid any distress, the VA will not mention anything about depression or other challenges that older adults may be facing. Older adults can use the app independently, but it is also beneficial for families, caregivers, and assisted living staff to share the mobile app with the older adult they care for. Overall, GoodTimes is a convenient and effective tool that can be used anytime, anywhere, with or without caregiver support, for cognitively intact older adults. This user study indicates that after using this app, users reported engaging in enjoyable conversations with the app.

## Methods

### Ethics Approval

This study was reviewed and approved by the institutional review board of NDSU. The IRB Protocol number is IRB0004419.

### Overview

**Figure 1:**
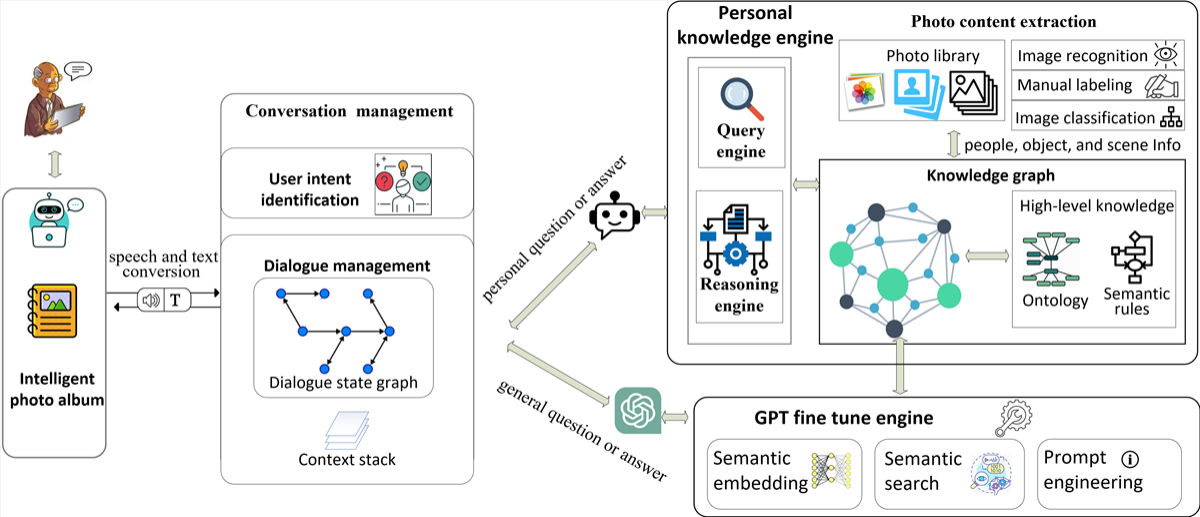
System Architecture.

GoodTimes is built on artificial intelligence (AI) technologies, including image recognition, natural language processing, knowledge graph, logic, and machine learning, to provide an interactive and personalized experience for older adults. The system architecture is illustrated in Figure 1. The app can be accessed through smartphones and tablets, and users can interact with it using their voice or fingers. The VA, which uses automatic speech recognition and natural language understanding technologies, converts the user’s voice into a text request. The conversation management module then processes the user’s request through 2 steps: user intent identification and dialogue management (DM). First, the user intent identification matches the user’s text request with predefined intents and dialogue states to create an input frame. Then, the DM module executes the dialogue policy based on the dialogue state graph and updates the dialogue state.

There are 2 types of communication requests: personal requests, which involve questions and answers related to the user’s personal information, and general requests, which do not necessarily involve personal information. Examples of personal questions include “Who is in the photo?” and “What is the story behind this photo?” General requests include knowledge not necessarily related to the user’s personal information, for example, a commonplace or a piece of widely known artwork. For instance, the AI may provide an answer about art collections in the Louvre Museum in Paris.

To handle general requests, we fine-tuned a Generative Pre-Trained Transformer (GPT; OpenAI) [16], a powerful neural language model. For personal requests, we have designed our own personal knowledge engine to provide tailored responses. Backend services of our personal knowledge engine are requested based on the user’s intent, and these services are supported by a semantics-based query engine and reasoning engine. These engines work over a knowledge graph, which is the brain of the system. The knowledge graph contains facts, relationships, and rules about photos, people, places, time, and stories. The search and reasoning engines link the dialogue with a specific photo, user profile, and context to enable personalized services. Finally, the DM module generates responses using the speech act and content selected based on the input frame.

The system maintains a library of photos that can be uploaded by the older adult user’s family members and caregivers. Metadata of a photo, such as people, animals, location, time, and special events, are also saved in the knowledge. Family members get involved in uploading, sharing, and explaining the photos, promoting collaboration, and having fun. Involving family members in the photo album can improve their relationship, help family members learn more about older adults, and facilitate reminiscence interventions for older adults, with or without caregiver support.

### Constructing an Open Personal Knowledge Graph

**Figure 2:**
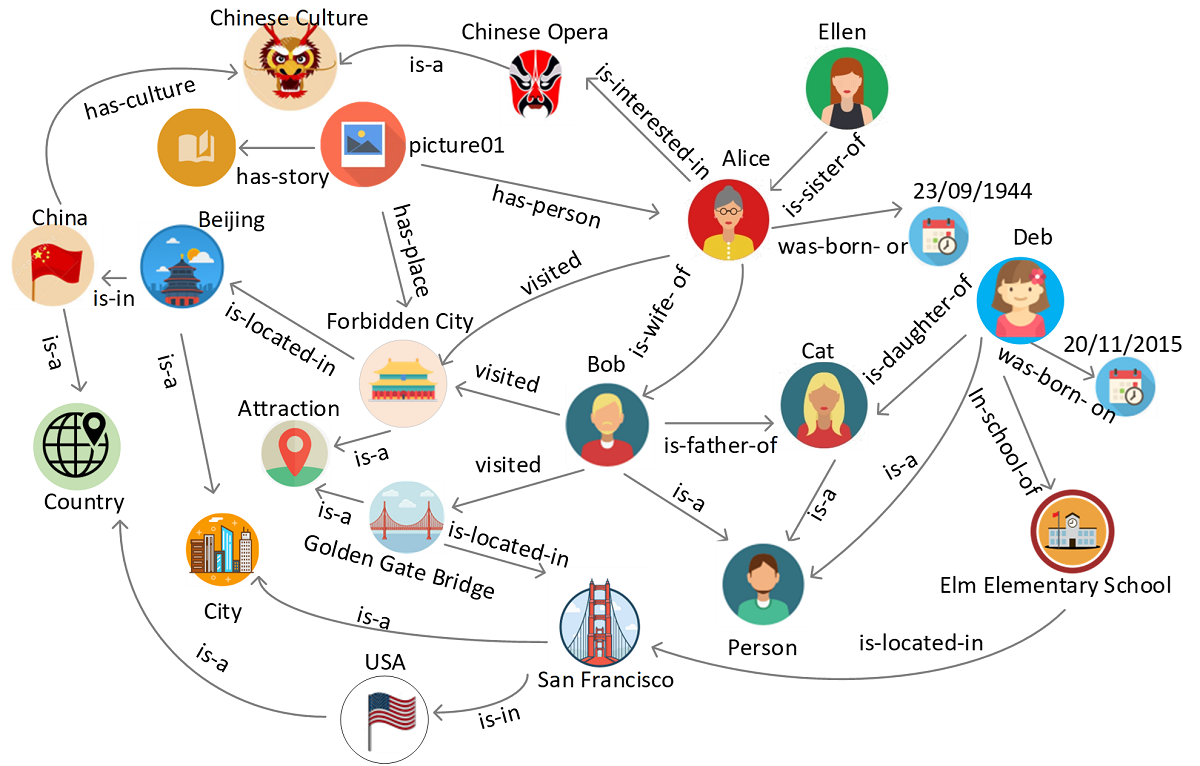
An example of a personal knowledge graph of user Bob.

The “brain” of the system is a comprehensive knowledge graph [17] that contains knowledge about the user and the photos. All the knowledge is represented as a graph in which data is modeled as nodes (vertices) and links (edges) between them. Nodes in our knowledge graph are normally a person, place, location, or thing, and links are how they are connected or related. Figure 2 shows an example knowledge graph about an older adult, Bob. In this graph, Bob, Alice, Cat, Person, Attraction, and Golden Gate Bridge are nodes. They are connected by many different relationships. For example, Alice is Bob’s wife, and Bob is the father of Cat. Alice, Bob, and Cat are persons. Bob visited the Golden Gate Bridge, which is an attraction. This example graph shows many things and relationships about Bob. It is used by the system to explain the story of the picture, including the people inside the picture, their relationships, where they visited, etc.

Graphs are used to model knowledge because they can encode real-world knowledge as “things” (not “strings”) and their interrelationships. This enables the system to communicate with users by analyzing what the words in a sentence actually mean rather than simply analyzing strings of characters. In addition, structuring knowledge in the form of graphs allows knowledge to evolve over time. New “things,” relationships, and external knowledge can be easily added or linked to the existing graph. In our system, we extend the core knowledge graph with external knowledge graphs, such as Wikidata [18,19], to extend our knowledge. Furthermore, reasoning and navigation can be performed over knowledge graphs.

#### Knowledge Graph Construction

**Figure 3:**
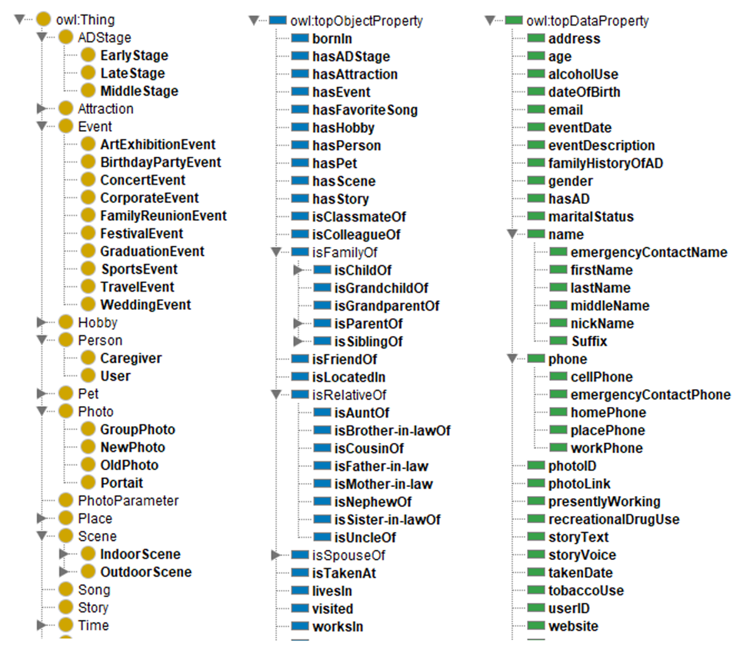
Part of the photo album ontology.

First, we built a high-level ontology working as the schema of the knowledge graphs. Then, we create a knowledge graph by instantiating the ontology with detailed instances retrieved from uploaded photos (with metadata) and user surveys and external knowledge graphs, such as Wikidata. Using ontology would allow logical inference for retrieving implicit knowledge rather than only allowing queries requesting explicit knowledge. We proposed a “Who-What-When-Where” model as the foundation of this album ontology. “Who” represents the person in or not in the photo but related. “What” points to the story related to a photo. “When” specifies the time when the photo was taken, which can be a date, a social occasion, or a historical monument. “Where” describes the place where the photo is taken. Figure 3 shows a major part of the ontology. This ontology is instantiated with instances through photo metadata extraction, tagging extraction, image recognition, and social media extraction.

The ontology serves as schema-level knowledge used to instantiate instances or individuals, thereby creating a comprehensive knowledge graph. Instance information is collected through various means, such as automatically extracting metadata from photos or through manual input or voice-based question and answer. Through these processes, a detailed knowledge graph like the one shown in Figure 2 can be generated.

#### Semantic Rule Definition

In order to facilitate logical inference and gain new insights from the knowledge graph, we have established a set of rules and used logical reasoning over the ontology. We provide a few examples of these rules below, noting that some are specified within the ontology itself, while others are created separately using Semantic Web Rule Language [20-22]. For clarity, we present all rules using the same first-order logic [22] format.

Relatives:

If x is the husband of y, then y is the wife of x, and vice versa:

∀x,y Husband(x,y) ⇔ Wife(y,x)

If x is the mother or father of y, then x is also the parent of y, and vice versa:

∀x,y (Mother(x,y) ∨ Father(x,y)) ⇔ Parent(x,y)

If z is the parent of both x and y, and x is not the same as y, then x and y are siblings:

∀x,y,z (Parent(z,x) ∧ Parent(z,y) ∧ x≠y) ⇒ Sibling(x,y)

If x is the parent of y, and y is the parent of z, then x is the grandparent of z:

∀x,y,z (Parent(x,y) ∧ Parent(y,z)) ⇒ Grandparent(x,z)

Social Relations:



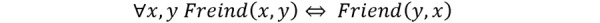





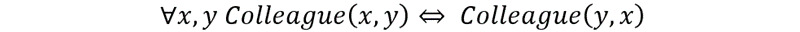





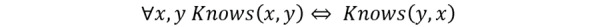



*…*

Time:

∀x,y,z Time(x) ∧ Time(y) ∧ Time(z) ∧ Before(x,y) ∧ Before(y,z) ⇒ Before(x,z)

∀x,y Time(x) ∧ Time(y) ∧ Before(x,y) ⇒ After(y,x)

*…*

Location:



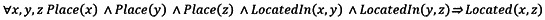



*…*

Photo co-occurrence Relations:

//Person p visited location l at time t, and took a photo ph

∀p,l,t,ph (Person(p) ∧ Location(l) ∧ Time(t) ∧ Photo(ph) ∧ PersonInPhoto(p, ph) ∧ PhotoTakenAtLocation(ph, l) ∧ PhotoTakenTime(ph, t)) ⇒ Visited(p, l, t, ph)

//Person p1 and p2 visited location l at time t together

∀p1,p2,l,t,ph Person(p1)∧Person(p2) ∧ Visited(p1,l,t,ph) ∧ Visited(p2,l,t,ph) ⇒ VisitedTogether(p1,p2,l,t)

### Dialogue Management

The photo album’s VA is a machine learning-based system that enables users to engage with it through natural conversation. The VA is capable of understanding user intents from free text, answering questions, and asking questions for a specific purpose. To promote reminiscence intervention, GoodTimes guides users in recalling their memories by asking them photo-related questions and responding based on their answers. The VA facilitates personalized questions during the dialogue flow and can route natural conversations with users. A photo album with a VA can help older adults feel more connected to their past and present, providing them with a sense of familiarity and comfort, which is especially important for older adult users. Our dialogue flow management includes the following key points to facilitate engaging interactive conversation: VA-driven conversation, intent recognition, context management, personalization, and empathy incorporation.

#### VA-Driven Conversation

In our app, the VA initiates and guides the conversation. The conversation between the VA and the user begins with a friendly greeting or prompt, followed by a series of questions that revolve around the “Who-What-When-Where” themes but are not limited to them. These questions are designed to elicit specific information about the photos from the user and jog their memory. We use techniques including contextual prompts, confirmation prompts, and error handling to let the VA control the dialogue flow.

The VA provides prompts or suggestions to the user based on the current context of the conversation. For example, if the user talks about a specific photo, the VA can suggest related topics or questions to keep the conversation flowing smoothly. The VA uses confirmation prompts to confirm the user’s intent or response to a question. This is useful when the VA needs to verify information before moving on to the next question or action. When the user provides incorrect or invalid input, the VA will provide appropriate responses, including rephrasing a question or prompt, asking for clarification, or providing an explanation of what the VA is looking for. By using these techniques, the VA can guide the conversation in a way that ensures the user provides the necessary information while keeping the conversation under control.

#### Intent Recognition

There are 2 ways to identify the user’s intent. One is to fine-tune a GPT to let it specify the intent or use our designed intent identification model (IIM). In our implementation, we used our own IIM as the main method, as GPT is more expensive. In our IIM, the VA uses the natural language processing algorithm, part of speech tagging [23], to break down a sentence or phrase into its constituent parts, such as nouns, verbs, and adjectives. Then, it uses named entity recognition [24] to extract important information such as the user's intent, entities (relevant keywords or phrases), and context from these components. We use machine learning algorithms (eg, our previous proposed algorithm [25]) to analyze the user’s input and match it with the most relevant intent. To train the model, we provide sample user inputs and assign them to specific intents. The VA then uses these examples to learn patterns in the data and improve its ability to recognize user intent over time.

#### Context Management

The VA also keeps track of the conversation’s context, including previous statements made by the user and the VA’s responses. This helps to ensure that the VA’s responses are relevant to the current conversation. Context management in the VA of the interactive photo album is critical to providing a seamless and personalized user experience. VA uses a context stack to manage the context of the conversation. For example, suppose the user is looking at a photo of a trip to Paris taken in front of the Louvre Museum. In that case, the VA can use this information to provide related suggestions or ask follow-up questions, such as “Did you see Leonardo da Vinci’s Mona Lisa in Louvre?” These questions are generated by prompting GPT using our knowledge graph and previous conversation history as context. The VA also needs to be able to handle changes in context, such as if the user switches to talking about a different topic. In such cases, the VA must recognize the change in context and adjust its responses accordingly. Overall, effective context management is crucial to creating a personalized and engaging experience for users interacting with the VA in the interactive photo album.

#### Personalization

The VA personalizes the conversation by considering multiple factors, including the user’s preferences, personal profile such as name, age preferences, and conversation history. The very basic form of personalization is addressing the user by name to make the conversation more personal and engaging. In addition, the VA will use knowledge in the knowledge graph to address people or things in the photo. For example, the VA will use the information stored in the knowledge graph to refer to people or things depicted in the photos. For instance, if the user’s mother is shown in the photo, the VA may address her as “your mother, Susan,” as her name is known from the knowledge graph. Similarly, if the user's pet dog is in the photo, the VA may refer to the dog by its name, “Buddy.” Additionally, suppose the knowledge graph indicates that the user has a close relationship with a particular person. In that case, the VA can refer to them with a personal term, such as “your dear friend, John.” This personalization can enhance the user’s experience and create a more natural and engaging conversation. Also, the VA uses the conversation history to tailor the conversation. For example, if the user has previously shown a preference for a particular type of photo or event, the VA can use this information to recommend similar photos or events.

#### Empathy Incorporation

The VA incorporates empathy into dialogue to create a more natural and engaging conversation. This involves understanding and responding to the user’s emotional state, using appropriate tone and language, and showing concern for the user’s needs and feelings. Older adults and people with Alzheimer disease may have difficulty understanding complex sentences or abstract concepts [26]. Our VA uses simple, clear language to make sure they understand what the VA is saying. The VA always tries to be patient and understanding when asking older adults about photos. The VA gives positive feedback when the user answers questions correctly or remembers important information. If they provide incorrect answers, the VA will gently correct them and provide additional context or information. It is also important to repeat questions if they are not answered correctly, as older adults may need more time to process and remember information. Asking related questions, such as about memories of a trip shown in the photo or the hobbies of a person in the photo, can also be helpful in stimulating memories and encouraging conversation. Overall, we try to create a comfortable and positive environment for older adults to share their memories and stories.

### Conversation Using Knowledge Graph

The knowledge graph is the source of information for conversing with users and is stored in Neo4j [27-29], a graph database that the VA uses to ask and answer questions about photos. Cypher [27-29], Neo4j’s query language, is used by the VA to navigate the graph and generate questions and responses. Natural language queries and answers from users are converted into Cypher queries. For example, if a user asks, “Who is in this photo?” the VA can convert this into a Cypher query that retrieves all people in the photo. To generate photo-related questions, Cypher first locates a specific photo node based on certain criteria. Relevant properties are then extracted from this node to generate a question, with the property value serving as the standard answer. Cypher can also query for a relationship linked to this node, creating a directional triple consisting of the photo node, a relationship, and another node. The knowledge graph uses directional triples (node-relationship-node) to express semantic data as subject-predicate-object. For more complex questions with multiple bindings, Cypher enables searching for nearby nodes and relationships by limiting the number of hops and specific relationships.

To keep the conversation engaging, the VA can ask follow-up questions related to the previous topic. For instance, if the previous query was about Mary, the VA might ask, “Do you remember Mary’s hobby?” by using the previous query result (ie, Mary) as the subject or object of a new triple. The new question is generated based on this triple, with the other node or relationship serving as the question and the remaining element in the triple as the standard answer. In addition, GPT [30,31] is used to generate related questions and content, which will be discussed further later on.

### GPT as a Complement

GPT is the state-of-the-art large language model used for various natural language processing tasks, including “question answering.” One of the main advantages of GPT is its large amount of pretrained knowledge, which enables it to understand and generate natural language text with high accuracy. However, the cost of using GPT could be prohibitive for some applications, including our system. Despite this limitation, we still benefited from GPT’s capabilities by using it to provide complementary functions. For example, we used GPT to help identify user intent if our IIM was not confident about its result. Moreover, GPT was used to enhance the conversational experience by generating follow-up questions and responses. For instance, if a user asks a question about a particular topic, GPT can generate related questions or statements that may help the user explore the topic further.

To ensure that the responses generated by GPT are relevant and accurate, we provided appropriate context for the conversation. One way to achieve this is by leveraging our local knowledge graph and conversation history as a source of relevant information. By converting the knowledge graph database into a triple format (subject, predicate, and object), we could index the triple file using semantic embeddings, which represent text data in a continuous vector space. This allows for efficient comparison and retrieval of similar text items while preserving semantic relationships between words and phrases. To generate embeddings, we used the pre-trained embedding model “Bidirectional Encoder Representations from Transformers” [32], resulting in a single vector embedding. These embeddings were then used in semantic search, enabling efficient and cost-effective searching. When a user query was received, we converted it into semantic embeddings and matched them with the embeddings of the knowledge graph using cosine similarity. This helped us identify the most relevant or related information sources in our knowledge graph that are related to the user’s query. Once we had identified the most similar embeddings in our local knowledge base, we used them as context or query input for the GPT model. This context provides GPT with relevant information from our local knowledge base, allowing it to generate relevant responses. In summary, while GPT may not be used extensively in our system due to its cost, it can still provide valuable complementary functions that enhance the user experience and help us better understand the user’s intent.

### Prototype System

We have implemented GoodTimes as an Android-based app using a mobile app development framework called Flutter [33]. The app includes a VA that uses Google’s Dialogflow [34] to understand natural language. When the VA identifies what the user wants, it uses Google Cloud Functions to interact with a backend server built with Spring Boot [35]. This server connects to a Neo4j database that stores information in the form of a knowledge graph. We used *LangChain* (Harrison Chase) [36], a Python package, to integrate OpenAI’s GPT language models with the knowledge graph. Specifically, we used the GPT-3.5 Turbo model and defined the GPT language model using the LLMPredictor class and the input prompt format using the PromptHelper class. The user has the option to interact with the app using either voice commands or touch input.

### Use-Case Study

We conducted a use-case study to evaluate the functionality of our interactive photo album app before deploying it to real users. This involved identifying and analyzing specific scenarios in which the app could be used, such as browsing photos, sharing them with friends and family, and asking questions related or unrelated to the photo. By examining how the app was used in these scenarios, we were able to identify areas for improvement to make it more user-friendly. The creation of fictional users for the use cases involved careful consideration of relevant characteristics and demographics aligned with this study’s objectives. Factors taken into account included age, gender, cultural background, language proficiency, cognitive and physical abilities, technological familiarity, and more. This iterative process allowed for refinement based on the specific goals and requirements of our research or design project.

Let us consider a fictional character, Bob, who is 79 years old and has memory challenges that affect his ability to remember things. As part of our use case, we presented Bob with a collection of photos encompassing significant moments such as his granddaughter Sara’s birthday party, his memorable trip to Paris with his wife, and heartwarming memories with his beloved pet dog, Lucy.

**Figure 4:**
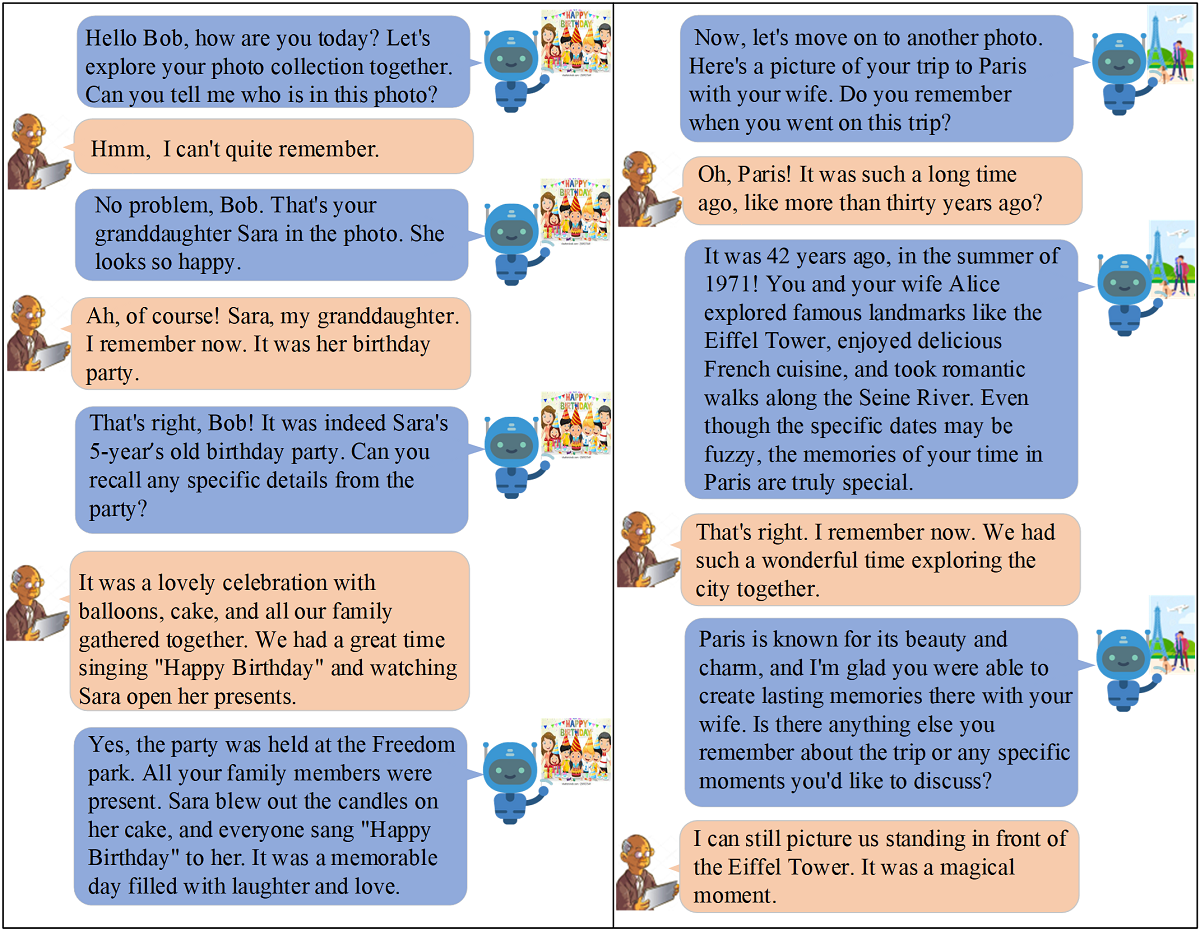
An example case of communication between the voice assistant (VA) and a user.

Figure 4 exemplifies the interaction between Bob and the VA, showcasing their communication regarding photos in the album. This particular example demonstrates the VA’s capability to engage with Bob and help him remember significant moments. In the conversation, Bob and the VA discuss a photo from Sara’s birthday party. Initially, Bob is unsure of the people in the photo. The VA kindly reminds him that it is Sara in the picture, which prompts Bob’s memory. The VA provides additional information about the event, refreshing Bob’s memory and filling in the gaps. The VA also helps Bob recall his trip to Paris. This interaction highlights the VA’s ability to serve as a helpful reminder and facilitator of memory recall. By engaging in conversations and providing contextual information, the VA helps Bob reminisce about important occasions and people in his life. This feature demonstrates the valuable role the VA plays in aiding individuals with memory challenges by providing gentle reminders and support.

In addition to the previously mentioned use cases, we conducted evaluations on 12 additional scenarios for our app. The diverse range of use cases demonstrated the app’s potential as a valuable tool for helping older adults cherish their memories and enhance their overall well-being.

### User Study

We conducted a user study to evaluate the usability and features of the GoodTimes app. Participants were recruited through convenience sampling using various digital communication channels, such as phone calls, social media, and email. To accommodate participants’ preferences, the study sessions were conducted in person at locations including their homes, local coffee shops, or parks. These sessions were conducted individually, allowing for personalized interaction and feedback.

At the beginning of each session, participants were provided with an overview of the study objectives and the app’s key features. Written consent was obtained from each participant, following the approved study protocol by the institutional review board of North Dakota State University. A video demonstration was presented, and participants were given approximately 30 minutes of hands-on practice with the app. A researcher was present to address any questions during the session. Following the interaction, participants completed a set of questionnaires to provide feedback on their experience, which took approximately 15 minutes to complete. This approach enabled us to gather comprehensive insights into the app’s usability and user experience.

The questionnaire consists of 2 main parts. The first part focused on participants’ experiences with the app’s features, including their interactions with the intelligent agent and their emotional responses to the digital photo gallery. They rated their experiences with 5 statements on a Likert scale ranging from “strongly agree” to “strongly disagree.” The survey included questions developed by the research team based on previous research and expert opinions.

The second part focused on the overall usability of the app, and open-ended questions were used to identify areas for improvement. In this part, we used a subset of the System Usability Scale (SUS) to assess the app’s usability. The SUS is a widely recognized and validated instrument for evaluating the usability of interactive systems. We selected 5 relevant SUS questions out of a total of 10 and included them in our survey.

In our evaluation of the app's usability and user experience, we used statistical analyses to objectively understand participant feedback. Specifically, we used a 1-sample 2-tailed *t* test, a parametric test used to determine if the sample mean significantly differs from a known or hypothesized population mean. Given that our data was interval in nature and derived from Likert-scale responses, the 1-sample *t* test was particularly apt. The hypothesized population mean in our context was the neutral point on our scale, allowing us to discern whether participants’ responses significantly leaned toward agreement or disagreement.

## Results

### Participants

The user study included a total of 15 participants, with 13 of them completing the survey. Out of the 12 studies conducted, 11 were in-person sessions, while 1 was conducted on the web through Zoom (Zoom Video Communications) as per the participant’s preference. The participant demographics are summarized in Table 1. Among the participants, 8 fell in the age range of 55-64 years, 2 were in the age range of 65-74 years, and 3 were in the age range of 75-84 years. In terms of gender distribution, there were 8 male participants and 5 female participants. Regarding education, 3 participants held a PhD degree, 4 participants had a master’s degree, 3 participants had a bachelor’s degree, 1 participant had a college degree, and 2 participants had completed high school.

**Table 1:** Demographic information of the participants.

Variable		Participants, n (%)
**Age (years)**		
	55-64	8 (62)
	65-74	2 (15)
	75-84	3 (23)
	≥85	0 (0)
**Gender**		
	Man	8 (62)
	Woman	5 (38)
**Highest educational degree**		
	PhD	3 (23)
	Master’s	4 (31)
	Bachelor’s	3 (23)
	College degree	1 (8)
	High school	2 (15)
	Less than high school	0 (0)

### Evaluation Outcomes

In order to evaluate the GoodTimes app, we conducted a user study with 13 older adult participants to collect feedback on their experiences with the app and its usability. Table 2 encapsulates participants’ feedback on their conversational experiences with the VA. The outcomes revealed an overwhelmingly positive response to their interaction experience with the VA. A significant majority of participants (12 out of 13) endorsed the efficacy and relevance of the VA in their conversations. Specifically, 8 participants were in strong agreement that the VA disseminated pertinent information, while the remaining 5 concurred with this sentiment. Furthermore, a compelling majority (11 out of 13) expressed strong affirmation regarding the VA’s accuracy in sharing memory-related information. In terms of the VA’s language clarity and appropriateness, 12 participants were either in strong agreement or in agreement, indicating near-universal approval.

The *P* value, a measure of statistical significance, provides robust statistical validation of these findings. For instance, the statement “The VA provided relevant information” had a *P* value of <.001, suggesting that the observed results are extremely unlikely to have occurred by chance alone. Similarly, the almost identical *P* values for “The VA provided correct information” and “The VA’s language is appropriate and easy to understand” (both *P*<.001) underscore the authenticity and significance of these findings. A *P* value below the typical threshold of .05 indicates a significant difference from the expected neutral response. This provides strong evidence that participants genuinely felt the statements were accurate descriptors of their experiences.

Table 3 depicts a profound capacity to foster positive emotions and reminisce among the study participants. It was noteworthy that every participant either agreed or strongly agreed that the app invoked cherished memories. Moreover, a substantial majority signaled their agreement or strong agreement with the app’s efficacy in reviving memories of dear ones, such as friends and family. Impressively, a significant 11 out of 13 participants articulated that the app augmented their happiness.

In Table 3, *P* values offer compelling evidence of these perceptions. For the statement “Brings a lot of Good Memories,” a *P* value of <.001 indicates an exceptionally significant result, suggesting the overwhelmingly positive feedback was not a mere coincidence. The sentiment “accelerates thinking about friends and family” also received a *P* value of <.001, reinforcing the strong affirmation of the app’s ability to stir memories of loved ones. Furthermore, the feedback “Makes me happy” also manifested a *P* value of <.001, emphasizing that a significant number of participants derived joy from the app’s use. These *P* values, being well below the conventional .05 threshold, bolster the claim of the app’s potent capability to enhance emotional health through memory stimulation.

During the usability assessment phase of our survey, we gauged the app’s interface using standardized usability prompts. Table 4 depicts an overview of the results on app usability. The results elucidated a prevailing sentiment of approval among respondents concerning the app’s usability. Notably, a significant desire was expressed to engage with the system regularly, as indicated by a *P* value of .005, which suggests this sentiment was not merely by chance. Additionally, the system’s helpfulness and its design simplicity garnered significant endorsement, as evidenced by the compellingly low *P* values of <.001, respectively. This denotes a genuine appreciation for the system’s functionality and design among users.

**Table 2:** Participants’ feedback on their conversational experience with the voice assistant (VA).

Statement	Strongly agree, n	Agree, n	Neutral, n	Disagree, n	Strongly disagree, n	Mean (SD)	2-tailed *t* (*df*)	*P* value
Conversation was pleasant	8	3	1	1	0	4.3846 (0.9608)	5.1959 (12)	<.001
Conversation was fluent and natural	4	7	1	1	0	4.0769 (0.8623)	4.5029 (12)	<.001
The VA provided relevant information	8	5	0	0	0	4.6154 (0.5064)	11.5016 (12)	<.001
The VA provided correct information	11	2	0	0	0	4.8462 (0.3755)	17.7272 (12)	<.001
The VA’s language is appropriate and easy to understand	11	2	0	0	0	4.8462 (0.3755)	17.7272 (12)	<.001

**Table 3:** Participants’ feedback on their emotional response to app usage.

Statement	Strongly agree, n	Agree, n	Neutral, n	Disagree, n	Strongly disagree, n	Mean (SD)	2-tailed *t* (*df*)	*P* value
Brings a lot of Good Memories	10	3	0	0	0	4.7690 (0.4385)	14.5455 (12)	<.001
Accelerates thinking about friends and family	10	2	1	0	0	4.6920 (0.6304)	9.6773 (12)	<.001
Makes me happy	5	6	1	1	0	4.1538 (0.8987)	4.6290 (12)	<.001

**Table 4:** Overview of results on the app’s usability.

Statement	Strongly agree, n	Agree, n	Neutral, n	Disagree, n	Strongly disagree, n	Mean (SD)	2-tailed *t* (*df*)	*P* value
I would like to use this system frequently	0	9	3	1	0	3.6154 (0.6504)	3.4115 (12)	.005
I think the system is very helpful	4	7	1	1	0	4.0769 (0.8623)	4.5029 (12)	<.001
I think the system design is very simple and easy to use	4	8	1	0	0	4.2308 (0.5991)	7.4073 (12)	<.001
I feel very confident about using the system	4	4	3	2	0	3.7692 (1.0919)	2.5400 (12)	.03
I think that I would need the support of a technical person to be able to use this system	1	3	2	6	1	2.7692 (1.1658)	0.7138 (12)	.49

Another commendable finding was the respondents’ confidence in using the app without external technical assistance. This was statistically supported by a *P* value of .03, reflecting a valid level of user self-assurance. However, the topic of needing technical support to operate the system did receive varied responses. Interestingly, the statement “I think that I would need the support of a technical person to be able to use this system” had a *P* value of .49, indicating that this sentiment was not statistically significant and could likely be attributed to random variability.

## Discussion

### Principal Results

We designed, developed, and tested an interactive photo album app called GoodTimes that uses AI technology to engage in conversations with users and tell stories about pictures, including family, friends, and special moments. The app was developed using state-of-the-art AI technologies, including image recognition, natural language processing, knowledge graph, logic, and machine learning. We constructed a comprehensive knowledge graph that models the information required for effective communication, including photos, people, locations, time, and stories related to the photos. We then developed a VA that interacts with users by leveraging the knowledge graph and machine learning techniques.

In order to evaluate the GoodTimes app, we conducted a use-case study to verify its various functions in different real-life scenarios. Additionally, we conducted a user study with 13 older adult participants to collect feedback on their experiences with the app and its usability. We found that the feedback from our participants was highly positive, with 92% (12/13) reporting a positive experience conversing with GoodTimes. All participants mentioned that the app invoked pleasant memories and aided in recollecting loved ones, resulting in a sense of happiness for the majority (11/13, 85%). Additionally, a significant majority found GoodTimes to be helpful (11/13, 85%) and user-friendly (12/13, 92%). Most participants (9/13, 69%) expressed a desire to use the app frequently, although some (4/13, 31%) indicated a need for technical support to navigate the system effectively.

### Limitations

Our research has some limitations that we aim to address in future work. First, we plan to increase family members’ active involvement in the process of using the app by making it easy and enjoyable for them to upload pictures and record their voices. Additionally, the findings may not be generalizable to both cognitively impaired and cognitively intact older adults due to the small convenience sample used in this study.

To better understand the potential of our app as a supplementary tool for reminiscence therapy, we plan to deploy the app to their homes or assisted living environments, allowing them to use it for an extended period of time. By doing so, we can observe the app’s impact over time and gain valuable insights into how it can provide emotional and mental stimulation to improve their quality of life.

### Comparison With Previous Work

Previous research has shown that reminiscence therapy, which involves the use of photos and videos to stimulate long-term memory, can improve the emotional well-being of older adults [5,37]. However, providing personalized reminiscence therapy can be challenging for caregivers and family members.

Various studies have investigated technology as a means of supporting reminiscence therapy for older adults. For instance, Chen et al [38] developed an app that used a lifelogging device to capture photos and videos and presented them in a timeline format, resulting in improved quality of life and cognitive function for participants. However, this approach is limited to recent events and can be inconvenient for users to wear many devices. Additionally, the mashup process requires significant caregiver involvement.

Another study by Tsao et al [39] developed an augmented reality app that allows users to interact with virtual versions of their memories. The app was found to increase participants’ sense of control and satisfaction with their lives. The study by Schoneveld [40] developed an AR photo album prototype to facilitate communication between a person with dementia and their caregiver, family member, or friend, aiming to evoke more details and elements of memory and contribute to additional discussion material. The prototype has shown positive results in low- and high-fidelity prototype testing with experts and proxy testers. Another study [41] created digital reminiscence and music therapies using prompts such as photos, videos, and music. The study focused on the rural population, which has reduced access to dementia care services.

Compared with these studies, our GoodTimes app uses AI technology to engage in conversations with users and tell stories about pictures, including friends, family members, and special moments. This personalized approach aims to provide a more engaging and emotionally supportive experience for older adults, requiring minimum efforts from caregivers. This study’s results showed that the app was well-received by participants, who found it helpful, easy to use, and enjoyable. In conclusion, while previous research has shown the benefits of reminiscence therapy for older adults, our GoodTimes app adds a new dimension to the field by using AI technology to provide a personalized and engaging reminiscence therapy experience.

### Conclusions

In conclusion, this study demonstrated the potential of the GoodTimes app to provide personalized reminiscence therapy to older adults, improving their emotional well-being. The use-case study and user study results showed that the app was well-received by participants and provided a helpful, easy-to-use, and enjoyable experience. Although this study has some limitations, such as the need for a larger sample size and a longer evaluation period, we plan to address these limitations in future work. Our GoodTimes app adds a new dimension to the field of reminiscence therapy by using AI technology to provide a personalized and engaging experience. Overall, we believe that the GoodTimes app has the potential to positively impact the lives of older adults and their families.

## Abbreviations

AI: artificial intelligence
DM: dialogue management
GPT: Generative Pre-trained Transformer
IIM: intent identification model
SUS: System Usability Scale
VA: voice assistant
